# Multilevel analysis of personality, family, and classroom influences on emotional and behavioral problems among Chinese adolescent students

**DOI:** 10.1371/journal.pone.0201442

**Published:** 2018-08-09

**Authors:** Jiana Wang, Shu Hu, Lie Wang

**Affiliations:** 1 Department of Social Medicine, School of Public Health, China Medical University, Shenyang, Liaoning, People’s Republic of China; 2 School of Humanities and Social Sciences, China Medical University, Shenyang, Liaoning, People’s Republic of China; University of Virginia, UNITED STATES

## Abstract

**Background:**

The classroom environment plays a potentially important role in shaping the emotions and behavior of adolescents. However, few studies have focused on this factor. The aim of this study was to explore the association between the classroom environment and emotional and behavioral problems among Chinese adolescents while also considering personality-and family-related factors.

**Methods:**

This cross-sectional study was conducted in November and December, 2009. A set of questionnaires, including the Strengths and Difficulties Questionnaire (SDQ), the Eysenck Personality Questionnaire (EPQ), the Family Environment Scale (FES), the Questionnaire on Teacher Interaction (QTI), and the Center for Epidemiological Studies- Depression Scale (CES-D), were distributed to participants. A total of 5,433 Chinese adolescent students (aged 9–18) and 244 classroom teachers in Liaoning Province were ultimately included in the study. Hierarchical linear modeling was used to explore the factors associated with emotional and behavioral problems.

**Results:**

Multilevel analyses revealed that adolescent emotional and behavioral problems significantly varied among classrooms. Although personality and family characteristics exerted the greatest influence on adolescents’ emotions and behavior at the individual-level, interactions between classroom teachers and students increasingly affected adolescents with respect to age at the class-level. A mild positive association was found between adolescents’ mental health problems and the mental health of teachers.

**Conclusion:**

This study focused on the classroom environment in order to understand Chinese adolescent mental health problems, the findings of which highlight important implications for policymakers and educators. The results underscore the importance of establishing a comfortable classroom climate by improving teacher-student interactions and meeting specific needs at different school stages, thus promoting a climate of positive mental health among Chinese adolescents.

## Introduction

Over the years, increasing emphasis has been placed on the importance of understanding adolescent emotional and behavioral problems [1], largely due to the growing prevalence of mental health problems among adolescents and young adults [2,3]. More than half of adult mental health problems emerge in early adolescence and contribute to the heavy burden of disease among young people as well as among those in later life stages [4]. Recent research suggests that poor mental health in adolescence is associated with teenage pregnancy, HIV/AIDS, crime, and suicide [5]. Adolescence represents a significant transitional period in terms of both physical and psychological development during which deviant behaviors and negative emotions may occur frequently, rapidly developing into firmly rooted problems, although early interventions have been shown to be particularly important and effective [6,7]. Therefore, it is important to identify the factors that are associated with adolescent problems in order to further the development of appropriate prevention programs and improve adolescent mental health.

It is widely accepted that behavior is a function of a person and his/her environment [8]. Personal factors, such as personality, have been commonly shown to impact the emotions and behavior of adolescents [9]. Similarly, the family environment and parental factors contribute significantly to the development of children’s mental health [10,11]. However, upon entering the school system, children transition from living primarily within their parents and family to spending most of their time in a classroom environment with teachers [12]. As such, the classroom constitutes an increasingly important environment during adolescence. Unfortunately, in contrast to the vast array of research examining family factors, only a limited body of research has explored the influence of the classroom environment on emotional and behavioral problems in adolescents. Amongst all aspects of the classroom environment, the interaction that occurs between teachers and students is especially important. Classes are communicative systems, thus highlighting the importance of interactions between teachers and students, and teachers adopt different styles of interaction with their students, which furthermore contributes to differences in students’ behavior [8]. Several studies have revealed that having a supportive teacher can foster good behavior [13,14] and is a protective factor against adolescents engaging in risky behaviors [12,15]. Some research highlighted school- and teacher-related influences on student mental health and carried out school-based interventions. The findings suggested that school-based trials could improve student mental health by focusing on teacher mental health [16]. Additional studies proposed that teachers’ skills could be harnessed for proactive behavior management and that the promotion of socio-emotional regulation might be more effective in improving and managing student mental health [17,18]. From a Chinese cultural perspective, the Chinese classroom represents the basic management unit within a school. As schools have fixed classrooms and teachers, students have the same teacher for several years, with the exception of special circumstances including, for example, students who transfer from another school or teachers who transfer from another job. Therefore, it is reasonable to propose that although this particular school system may lend stability to the student-teacher relationship, it exerts a continuous influence on student mental health, in excess of that observed in Western schools. In addition, China is generally a collectivist culture, whereby the rights of the individual are subordinated to those of the group. Collectivism emphasizes interdependence and the authority of the leader. Therefore, Chinese collectivist culture tends to value the role of the school and school teachers in child development [19], and being disapproved of and disliked by teachers might have a strong impact upon students’ behavior and mental health. All of these factors contribute to the profound impact that a classroom teacher may have on the socialization of Chinese adolescent students. A Chinese study revealed that the most important interpersonal relationship for students was with teachers rather than with parents and peers [20]. Unfortunately, few studies have focused on the classroom, and most studies, either in China or abroad, were carried out by examining just one grade or school stage, thus leading to difficulties in identifying and catering to the specific needs of adolescents at different school stages. Similarly, few studies have performed a reasonable analysis of nested data, which is necessary as students are nested in classes. Therefore, it was important to systematically investigate the factors that are associated with Chinese adolescent mental health, particularly those factors that are specific to classroom teachers, and to adopt a reasonable methodology as well as representative sample sizes to study the entire school period for the purpose of facilitating the design of primary care interventions in schools.

Notably, the personal psychological status of the teacher, which takes account of job stress, burnout, and well-being, has been considered an important predictor of child behavior [21]. Therefore, this study also attempted to evaluate the association between student behavioral problems and the mental health of the classroom teacher, noting mental health concerns such as depression. In addition, as the classroom environment has been considered to moderate the influence of some factors on student behavior [22,23], the current study further explored the moderating effect of the teacher–student relationship with respect to the association between adolescent personality and emotional and behavioral problems.

Based on the issues outlined above, this study examined the following hypotheses: (1) while both family environment and personality-specific differences account for much of the variation in mental health problems at the individual-level, the mental health of teachers and the average teacher–student interaction further explain this variation at the class-level; (2) the association between student problems and classroom teacher-related factors differs across school stages; and (3) the teacher’s interaction style moderates the association between adolescent personality and mental health.

## Materials and methods

### Ethics statement

This study obtained ethical approval from the Committee of Human Experimentation at China Medical University. Having explained the nature of the survey, written informed consent was obtained from each participant. Each participant’s privacy was safeguarded with respect to the processing of personal data and the confidentiality of individual records and accounts was ensured.

### Study design and study sample

This study was conducted in Liaoning Province, northeast China, during November and December, 2009. Three cities were randomly selected (i.e., one metropolitan city, one medium-sized city, and one small city) in both urban and rural areas. Six public schools were randomly selected from each area, including two primary schools (grades 4–6) and four senior schools (grades 7–12). One-third of the classes were randomly selected from each grade. According to the estimated sample size, 25 students in each designated class were randomly selected, with an equal number of boys and girls. Finally, a total of 6,975 students and 279 classroom teachers were chosen to participate in this study. All participants and their parents were thoroughly informed about the content and aims of this study. After having obtained written consent to conduct the survey, a set of questionnaires, including the Strengths and Difficulties Questionnaire (SDQ), Eysenck Personality Questionnaire (EPQ), Family Environment Scale (FES), and Questionnaire on Teacher Interaction (QTI) as well as a request for personal information were distributed to all 6,975 participants. An additional questionnaire, namely, the CES-D and a request for personal information were distributed to the 279 classroom teachers. A total of 6,658 students returned the questionnaires, 335 of which were excluded from the study. In addition, 24 of the 268 teachers who returned their questionnaires were excluded due to having provided incorrect information or missing data. A further 890 student participants were excluded due to incorrect information or missing data concerning their classroom teachers on the questionnaires. Comparing the students that were excluded with those students who were included, there were no significant differences (P>0.05) in gender and age (for gender: 43.6% of boys vs. 44.4% of boys; for age: 13.26 SD = 2.4 vs. 13.43 SD = 2.5. For SES, there were 30.8% in low level, 45.6% in middle and 23.7% in high among included students vs. 31.7% in low level, 42.4% in middle and 25.9% in high among excluded students. Although a significant difference (P<0.05) was found in SES, the percentage in each level was very similar. The excluded teachers were from various types of schools in each city size. Therefore, we have reasons to believe that the excluded students can match the included students in basic characteristics. The final sample size consisted of 5,433 Chinese adolescent students and 244 classroom teachers. The effective response rate was 77.9% for students and 87.5% for teachers. The procedures followed were in accordance with the ethical standards of the Committee of Human Experimentation at China Medical University.

### Measurement of emotional and behavioral problems

The self-report SDQ was utilized to identify emotional and behavioral problems [24]. The self-report SDQ includes 25 items, five scales of five items each: emotional symptoms, conduct problems, hyperactivity/inattention, peer problems, and prosocial behavior. The sum of all scales, except the prosocial behavior scale, produced a total score to assess emotional and behavioral problems, which represented the outcome variable. Higher scores denote more severe problems. The Chinese version of the self-report SDQ has been validated in China [25]. The Cronbach’s alpha was 0.70 for the total sample.

### Measurement of personality

Personality was assessed using an adapted version of the EPQ for children [26]. This version has 88 true-false items and includes four subscales: extraversion (E), psychoticism (P), neuroticism (N), and lie (L). The Cronbach’s alpha was 0.70 for the total scale, 0.76, 0.76, 0.88, and 0.77 for E, P, N, and L, respectively.

### Measurement of family environment

The FES has 90 true-false items and includes ten subscales: ‘cohesion’, ‘expressiveness’, ‘conflict’, ‘independence’, ‘achievement’, ‘intellectual-cultural orientation’, ‘active-recreational orientation’, ‘moral-religious emphasis’, ‘organization’, and ‘control’ [27]. Seven subscales were used in this study as the remaining three subscales were deemed inapplicable with respect to the Chinese population (i.e., ‘expressiveness’, ‘independence’, and ‘moral-religious subscales’) [28]. Cronbach’s alpha was 0.72 for the total scale and 0.74, 0.65, 0.43, 0.58, 0.67, 0.64, and 0.55 for each respective subscale.

### Measurement of teacher–student interaction

Teacher–student interaction was measured using the QTI, which was developed to map interpersonal teacher behavior according to eight subscales: (1) leadership–the teacher provides leadership to the class and holds the attention of students; (2) helpful/friendly–the teacher is friendly and helpful towards students; (3) understanding–the teacher shows understanding and care to students; (4) student responsibility/freedom–the students are given opportunities to assume responsibilities for their own activities; (5) uncertainty–the teacher exhibits her/his uncertainty; (6) dissatisfaction–the teacher shows unhappiness/dissatisfaction with the students; (7) admonishment–the teacher displays anger/temper and is impatient in class; and (8) strictness–the teacher is strict and is demanding of students. This study utilized students’ responses from the shorter 48-item Chinese revised version of the QTI (i.e., six items for each scale), which was appropriate for primary students [29]. It has been widely used among Chinese populations and has good reliability and validity [30]. Cronbach’s alpha was 0.73 for the total scale at the student-level and 0.65, 0.80, 0.41, 0.67, 0.76, 0.50, 0.82, and 0.65 for the eight respective subscales. According to the research of Ludtke,et al.[31], intraclass correlations (ICCs) were calculated to assess the classroom reliability, and the reliability of QTI was 0.78 for the total scale and 0.86, 0.88, 0.86, 0.77, 0.80, 0.89, 0.89 and 0.78 for the eight respective subscales.

### Measurement of depressive symptoms

The Chinese version of the CES-D contains 20 items [32]. In this study, the Cronbach’s alpha was 0.89.

### Confounding factors

Confounding factors included students’ gender, age, and socioeconomic status (SES) as well as classroom teachers’ gender and age. SES was determined by the parents’ highest education level and was divided into low, middle, and high SES.

### Statistical analysis

#### Overview of hierarchical linear modeling (HLM)

The traditional linear model, often used in previous class studies, requires that variables are mutually independent. However, in the present study, students were nested in classes. Thus, students’ emotions and behaviors were not independent from those of their classmates. Both the translation of class-level data to the individual-level and the combining of individual-level data with class-level data may produce an inaccurate analysis or lead to the loss of important sample information. The class, rather than the student, should be used as the main unit to analyze class factors. HLM is an ideal model that can be utilized to evaluate two-level nested data as it does not require the variables to be independent.

#### Analytic process

All data analyses were performed separately for primary, junior, and senior high school groups. First, it was established that the null model contained no predictor variables. The intraclass correlations (ICCs) were calculated for the SDQ and the chi-square test was used to verify significant differences in the SDQ responses between classes. As such, a two-level model was deemed appropriate. This model divided the total variance into individual and class components and estimated the proportion of the variance that could be attributed to each level. The complete model, including the individual-level variables (subscales for the EPQ and FES, and students’ fixed factors) and the class-level variables (subscales for the QTI and CES-D, and teachers’ fixed factors), was designed to predict the direct effects of all variables on the SDQ. Among the class-level variables, student responses from the QTI varied within classes and each classroom had a single QTI score, which was the average score of all students within classes. Other variables, such as the CES-D and other information directly obtained from teachers, were constant at the class-level. This information as well as data derived from the QTI comprised the class-level database. The complete model included two components, namely, fixed components consisting of regression coefficients and t-test results, which were similar to the results obtained from ordinary least-squares regression. The direct effects of individual and class levels were mainly provided by the fixed model. The random components consisted of the variance and chi-square test results to estimate whether the regression coefficient of individual-level factors varied among classrooms. The random model provided clues to guide the next step. The individual-level factor, which showed a significant chi-square result, indicated that a cross-level interaction model was required. Finally, a cross-level interaction model was developed to explore the moderating effects of the QTI with respect to the association between the EPQ and SDQ. The cross-level interaction model was based on the complete model and included all factors, adding only the moderating effects. EPQ subscales that produced a statistically significant chi-square test result of random effects (p < 0.05) were added to the cross-level interaction model. All models were generated using HLM software (version 6.06). The descriptions of data were calculated using SPSS17.0.

In this study, the expressions of the basic form of HLM were as follows:

$$\begin{array}{l} {\mathrm{The}\;\mathrm{individual}\;\mathrm{level}:\;Y}_{\mathrm{ij}}{=\beta }_{0j}{+\beta }_{1j}X_{1\mathrm{ij}}{+r}_{\mathrm{ij}}\\ {\mathrm{The}\;\mathrm{class}‑\mathrm{level}:\;\beta }_{0j}{=\gamma }_{00}+\gamma_{01}W_{1}{}_{j}{+\mu }_{0j}\\ \;\beta_{1j}{=\gamma }_{10}{+\gamma }_{11}W_{1j}{+\mu }_{1j,} \end{array}$$

where X denotes subscales for the EPQ and FES as well as students’ confounding factors, while W expresses subscales for the QTI and CES-D, and teachers’ confounding factors.

## Results

The average age of adolescent participants was 13.43 years (SD = 2.5, range 9–18 years). This group included 2,412 boys (44.4%) and 3021 girls (55.6%). There were 92 classroom teachers in primary schools, 96 in junior high schools, and 56 in senior high schools, with an average age of 35.90 (SD = 4.9), 37.77 (SD = 5.7), and 34.90 (SD = 6.6) years, respectively. The characteristics of the students and classroom teachers as well as the differences between school groups are shown in Table 1. Table 2 illustrates the correlations between SDQ and all of the students’ analytical variables used in the regression model. P, N, ‘conflict’, ‘uncertainty’, ‘dissatisfaction’, and ‘admonishing behavior’ were positively correlated with SDQ for each school type (P<0.05). E, L, ‘cohesion’, ‘achievement’, ‘intellectual-cultural’, ‘active-recreational’, ‘organization’, ‘leadership’, ‘helpful/friendly’ and ‘understanding’ were negatively correlated with SDQ for each school type (P<0.05). Control was only negatively correlated with SDQ at primary school level. Student Responsibility/Freedom was only negatively correlated with SDQ at junior and senior middle school level. Strict behavior was only negatively correlated with SDQ at senior middle school level.

**Table 1:** Description of the SDQ, EPQ, FES, QTI, CES-D and sample characteristics in three school groups.

Variable	Primary (n = 2039)	Junior (n = 2091)	Senior (n = 1303)
	Mean ± SD		
SDQ			
Total difficulties score	9.62±5.33	11.19±5.80	13.12±5.81**
Emotional symptoms	2.14±2.09	2.76±2.35	3.43±2.37**
Conduct problems	1.89±1.52	2.30±1.60	2.71±1.73**
Hyperactivity/inattention	2.63±2.03	3.15±2.08	3.95±2.03**
Peer problems	2.97±1.53	2.98±1.61	3.03±1.65
Prosocial behaviors	7.65±2.16	7.76±2.07	7.69±2.07
EPQ			
Extraversion	18.39±4.29**	18.23±4.39	17.71±4.35
Psychoticism	2.49±2.65	3.14±2.97	3.85±3.18**
Neuroticism	5.97±4.55	8.08±5.68	10.25±5.67**
Lie	16.44±3.47**	14.93±3.79	12.73±3.82
FES			
Cohesion	7.92±1.56	7.70±1.80	7.36±1.98
Conflict	1.96±1.75	2.13±1.86	2.79±2.13
Achievement	8.73±1.39	8.61±1.45	8.42±1.52
Intellectual-Cultural	5.13±1.95	5.15±2.01	4.26±1.97
Active-Recreational	3.71±2.20	4.05±2.18	3.75±2.21
Organization	7.00±1.78	6.59±1.99	5.90±2.13
Control	4.20±1.95	3.61±1.84	3.32±1.78
QTI			
Leadership	19.93±1.46**	19.46±1.70	18.56±1.75
Helpful/Friendly	18.77±2.18**	18.67±2.50	16.99±2.37
Understanding	21.58±1.42**	20.97±1.88	19.59±2.08
Student Responsibility/Freedom	12.59±1.67	13.04±1.56**	12.78±1.61
Uncertainty	4.55±2.85	5.00±1.28	5.63±1.60**
Dissatisfaction	2.45±1.80	3.81±1.99	5.44±2.64**
Admonishing behavior	5.97±1.01	7.14±1.74	8.17±2.43**
Strict behavior	15.13±2.08	15.29±1.69	14.94±1.78
CES-D of classroom teacher	16.21±8.19	15.53±8.89	17.66±9.97
Demographic factors	**% (N)**		
Level 1: student			
Gender			
Male	44.8(914)	43.5(909)	45.2(589)
Female	55.2(1125)	56.5(1182)	54.8(714)
SES			
High	23.2(474)	27.1(566)	18.8(245)
Middle	43.6(889)	44.8(936)	49.9(650)
Low	33.2(676)	28.1(589)	31.3(408)
Level 2: class			
Teacher gender			
Male	8.7(8)	22.9(22)	51.8(29)
Female	91.3(84)	77.1(74)	48.2(27)

*P < 0.05

**P < 0.01

**Table 2:** Correlations between SDQ and all the student analytical variables in the regression model.

	SDQ		
	Primary (n = 2039)	Junior (n = 2091)	Senior (n = 1303)
E	-0.304**	-0.263**	-0.275**
P	0.520**	0.553**	0.518**
N	0.598**	0.661**	0.626**
L	-0.493**	-0.465**	-0.415**
Cohesion	-0.412**	-0.402**	-0.389**
Conflict	0.346**	0.387**	0.360**
Achievement	-0.095**	-0.103**	-0.089**
Intellectual-Cultural	-0.240**	-0.248**	-0.183**
Active-Recreational	-0.158**	-0.195**	-0.176**
Organization	-0.374**	-0.354**	-0.352**
Control	-0.056*	0.036	-0.031
Leadership	-0.188**	-0.241**	-0.253**
Understanding	-0.213**	-0.265**	-0.269**
Uncertainty	0.179**	0.266**	0.243**
Admonishing behavior	0.208**	0.257**	0.244**
Helpful/Friendly	-0.203**	-0.272**	-0.226**
Student Responsibility/Freedom	-0.023	-0.110**	-0.084**
Dissatisfaction	0.335**	0.349**	0.307**
Strict behavior	-0.003	-0.039	-0.095**

E: Extraversion; P: Psychoticism; N: Neuroticism; L: Lie

*P < 0.05

**P < 0.01

The results of the null model are shown in Table 3. The chi-square tests produced statistically significant results for all three school groups, indicating that there were significant differences in the SDQ results among classes and that the development of two-level models was warranted. The ICCs indicate the proportions of variance in the SDQ that is at the classroom level. The results from the complete model that tested the associated factors at both the individual and class levels for the three school groups are shown in Tables 4–6. Student variables containing the EPQ subscales (E, P, N and L), the FES subscales (‘cohesion’, ‘conflict’, ‘achievement’, ‘intellectual-cultural orientation’, ‘active-recreational orientation’, ‘organization’, and ‘control’), the fixed variables (gender, age and SES of students), class variables that consisted of the QTI subscales (‘leadership’, ‘helpful/friendly’, ‘understanding’, ‘student responsibility/freedom’, ‘uncertainty’, ‘dissatisfaction’, ‘admonishing’, and ‘strictness’), the CES-D, and the fixed variables (gender and age of teachers) were included in all of the regression models.

**Table 3:** The null model, including ICCs for the SDQ, for different school groups.

Statistics	Level	Primary (n = 2039)	Junior (n = 2091)	Senior (n = 1303)
Var. comp.	Individual	25.184	30.356	30.160
	Class	3.432	3.411	3.984
ICC		0.120	0.101	0.117
χ^2^		354.749**	321.114**	213.525**

ICC = Var(Class)/Var(Class)+Var(individual)

*P < 0.05

**P < 0.01

**Table 4:** The full model in primary school (n = 2039).

	Fixed		Random	
	Coef. (SE)	T-test	χ^2^	var.
Level 1: student				12.039
E	-0.165(0.026)	-6.370**		
P	0.245(0.046)	5.313**		
N	0.465(0.024)	19.059**		
L	-0.243(0.030)	-8.182**		
Cohesion	-0.215(0.070)	-3.087**		
Organization	-0.178(0.062)	-2.859**		
Control	0.143(0.0450)	3.187**		
Level 2: class				2.021
Understanding	-0.434(0.179)	-2.423*		
Dissatisfaction	0.635(0.105)	6.066**		
CES-D	0.040(0.017)	2.374*		

*P < 0.05

**P < 0.01

Gender, age, and SES in level 1and teacher gender and age in level 2 were fixed in the model.

E: Extraversion; P: Psychoticism; N: Neuroticism; L: Lie; CES-D: Depressive symptoms

**Table 5:** The full model in junior high school (n = 2091).

	Fixed		Random		
	Coef. (SE)	T-test	χ^2^	Original var.	Conditional var.
Level 1: student				11.845	
Level 2: class				1.985	
Low dissatisfaction group					
Level 1: student					
E	-0.135(0.026)	-5.114**			
P	0.304(0.069)	4.409**			
N	0.495(0.025)	20.097**			
L	-0.210(0.042)	-4.972**			
Conflict	0.238(0.096)	2.476*			
Level 2: class					
Student Responsibility/Freedom	-0.548(0.182)	-3.005**			
High dissatisfaction group					
Level 1: student					
E	-0.139(0.037)	-3.725**			
P	0.240(0.085)	2.809**			
N	0.474(0.032)	14.682**	67.720**	0.00314	0.00133
Understanding	-0.154(0.055)	2.777**			
L	-0.169(0.039)	-4.350**			
Control	0.218(0.084)	2.605*			
Level 2: class					
Understanding	-1.259(0.518)	-2.430*			
Uncertainty	0.767(0.226)	3.400**			

*P < 0.05

**P < 0.01

Gender, age, and SES in level 1 and teacher gender and age in level 2 were fixed in the model.

E: Extraversion; P: Psychoticism; N: Neuroticism; L: Lie; CES-D: Depressive symptoms

**Table 6:** The full model in senior high school (n = 1303).

	Fixed		Random	
	Coef. (SE)	T-test	χ^2^	var.
Level 1: student				12.686
E	-0.260(0.024)	-10.764**		
P	0.314(0.061)	5.139**		
N	0.455(0.023)	19.645**		
L	-0.218(0.033)	-6.543**		
Cohesion	-0.195(0.085)	-2.287*		
Level 2: class				1.337
Uncertainty	0.319(0.156)	2.051*		
Helpful/Friendly	-0.468(0.131)	-3.578**		
Student Responsibility/Freedom	0.372(0.182)	2.039*		
Dissatisfaction	0.310(0.119)	2.607*		
CES-D	0.053(0.020)	2.651*		

*P < 0.05

**P < 0.01

Gender, age, and SES in level 1 and teacher gender and age in level 2 were fixed in the model.

E: Extraversion; P: Psychoticism; N: Neuroticism; L: Lie; CES-D: Depressive symptoms

In primary schools (Table 4) and at the student-level, all EPQ subscales as well as cohesion, organization, and control were significantly associated with SDQ. At the class-level, SDQ had significant direct associations with students’ perceptions of teachers’ understanding, dissatisfaction, and teachers’ CES-D score. The chi-square test, based on the random component of these variables, did not prove statistically significant at the class-level (p > 0.05). The variance components showed that, compared with the null model, the student-level model variance was reduced by 52.2% (i.e., variance components were reduced from 25.184 to 12.039). Similarly, the class-level variance was reduced by 41.1%.

In junior high schools, the unexpected finding that SDQ was negatively associated with admonishing behavior (coefficient = -0.332, p = 0.022) was contrary to current theory as well as our own understanding. In order to better understand this unusual association, interactions were assessed between ‘admonishing behavior’ and other class-level variables, and an interaction was found between ‘admonishing behavior’ and ‘dissatisfaction’. Each classroom had a single dissatisfaction score, which was the average score for all students in the class. Classrooms were divided into low and high dissatisfaction subgroups. HLM has a higher sample size demand at class-level. While there is no unified standard with respect to the sample size at the second level, a larger sample size at the class-level could enhance the overall accuracy of the results. Previous studies generally utilized a sample size of 40 or more participants [14,33,34]. Therefore, by referring to the median score for ‘dissatisfaction’, the current study divided the classrooms into two subgroups in order to ensure the largest sample size in each subgroup and to enhance the reliability and credibility of the results as much as possible. As such, classrooms were divided into low or high subgroups in cases where the dissatisfaction score fell below or above the median. The researchers then ran the junior high school model separately for both subgroups (Table 5). In the low dissatisfaction group, the SDQ showed a direct association with the EPQ and ‘conflict’ at the student-level as well as with ‘responsibility/freedom’ at the class-level. In the high dissatisfaction group, the EPQ and ‘control’ at the student-level as well as ‘understanding’ and ‘uncertainty’ at the class-level were directly associated with the SDQ. The chi-square test of the random components showed that the N slope was significant (p < 0.05). Therefore, a cross-level interaction model was developed and showed that only ‘understanding’ was significantly associated with the N slope equation. The student-level variance was reduced by 61.0%, and the class-level variance was reduced by 41.8%. The variance components of N decreased from 0.00314 to 0.00131, accounting for 57.6% of the variance.

The results from senior high schools (Table 6) showed that the EPQ, ‘cohesion’, ‘uncertainty’, ‘helpful/friendly’, ‘student responsibility/freedom’, ‘dissatisfaction’, and the CES-D score were significantly associated with the SDQ. The variance components were reduced by 57.9% at the student level and by 66.4% at the class-level.

## Discussion

The current study is one of the first to explore the contributions of class factors on emotional and behavioral problems among Chinese school adolescents aged 9–18 years at different school stages, namely, primary, junior, and senior high school levels. The ICCs obtained from the null model indicated that a large proportion of the total variance for adolescents’ emotional and behavioral problems was between students within classrooms. This finding is consistent with the hypothesis as well as with the results of previous studies that reported that individual student characteristics had the greatest influence on their behavior [15]. All aspects of personality were significantly associated with emotional and behavioral problems at each school stage, which is in accordance with the findings of previous studies [9]. Therefore, personality is an important factor to consider in terms of alleviating the mental health problems of adolescents. As expected, the influence of the family climate gradually decreased with the progression through school stages. ‘Cohesion’ and ‘conflict’, which represented the primary focus of previous studies, were highly associated with emotional and behavioral problems [35], suggesting that a harmonious family environment may also be important to improve adolescent mental health.

This study was mainly focused on the association between adolescent mental health and the classroom environment. Significant differences in student behavior were found among classes. In addition, the average teacher–student interaction and the mental health of classroom teachers further explained this variation after having controlled for teachers’ demographic characteristics. The classroom teacher exerts the greatest influence on the classroom environment as well as on student development [36]. Moreover, prior to attending college, Chinese students have a fixed classroom with a specially designated classroom teacher. Therefore, the behavioral pattern or mental status of classroom teachers can directly affect students’ emotions and behaviors. Several studies have shown that a positive teacher–student relationship is beneficial for adolescent mental health [37,38]. As such, building a stable and positive teacher–student relationship is necessary to enhance students’ emotional growth and mental well-being. Overall, the present study found that students perceived their teachers as displaying largely cooperative behavior rather than oppositional behavior during interactions, as described in Table 1. These results imply that Chinese school students had primarily positive perceptions of teacher–student relationships, which is consistent with previous studies that reported similar experiences of teacher–student relationships among primary and senior high school students in Hong Kong and Korea, respectively [39,40]. Previous studies also suggested that Asian students perceived their teachers to be more positive than those from other cultural groups [27,39,40]. This result may be attributable to the fact that in Asia, classroom teachers tend to be authoritative and responsible for their students. As such, teachers gain respect from students, which may thus promote a well-organized classroom environment. Furthermore, in line with the second hypothesis, the influence of classroom teachers on students gradually increased. In the primary school model, the variance components in the class-level model decreased from 3.432 to 2.021 i.e., a decrease of 41.4%. In junior high school, the reduction in class variation was 41.8%, according to the difference value of variance components from 3.411 to 1.985, which is similar to the value for primary school. However, in senior high school, the class variation reduction reached 66.4%, although the quality of teacher–student interactions gradually decreased, thus highlighting increasing importance of the classroom teacher’s role as students progress through higher school stages. According to previous studies, teacher interaction becomes more important for students as they grow older, although the quality of teacher–student interactions decreases with higher school stages [36]. Late adolescence has been considered to represent a particularly vulnerable life stage and requires further recognition by adults [41]. Adolescents attempt to be independent and experience more positive interpersonal relationships with adults. These facts exacerbate the difficulties that teachers experience when communicating with students who, as a result, likely feel that they experience negative interactions with teachers. These findings implied that teacher–student interactions are particularly important for older adolescents and that educators in high schools should pay greater attention to their students.

The strongest predictor of adolescent mental health problems was the students’ perception that teachers adopted a passive dissatisfied teaching style, which influenced adolescents at all school stages. An additional study found that teacher dissatisfaction was the only significant negative factor associated with students’ affective and cognitive outcomes [42]. Teacher dissatisfaction describes teachers who appear to be unhappy, impatient, and distrustful, who can provoke feelings of frustration in students and cause them to feel instant disgust and even aggression [43], which can subsequently lead to emotional and behavioral problems among students. Teachers with an understanding teaching style likely enable students to feel comfortable in class. Some studies have considered that emotional support and warmth are important to promote student mental health [38]. This association may be better understood when considering the results of the current study examining younger adolescents, the mental and emotional immaturity of the children, and their particular need for care and support from teachers. In the high dissatisfaction junior high school group, students’ perception of teachers’ understanding also decreased the negative association between student mental health and neuroticism. The present study furthermore found that a helpful/friendly interaction style may prove the most beneficial for student mental health in senior high school. The results suggest that teachers’ kindness was especially important to students who exhibited somewhat low levels of mental health. Similarly, older adolescents have been found to have more emotional and behavioral problems [44], which is supported by the results of the present study. Therefore, senior high school teachers need to provide further help to students to assist them in managing more serious problems, which may also be crucial in protecting them from associated risks. Interestingly, with respect to teacher responsibility/freedom, this study found two conflicting results. For junior high school students in the low dissatisfaction group, the results suggested that being offered the opportunity to make their own decisions may benefit their mental health, which is in accordance with previous studies [45]. However, the results for senior high school contradicted this finding. In fact, freedom may be detrimental to adolescent mental health. Previous studies have indicated that students preferred slightly stricter teachers who had less tolerance for poor behavior and better classroom organization [43, 46]. In addition, the senior high school students in the present study generally perceived the worst teacher–student interactions. This result suggests that teachers should maintain a balance between control and freedom, particularly in classes with poor teacher–student relationships in order to prevent indulging them of poor behaviour, which would only generate further problems. Behavioral problems among junior and senior high school students were positively associated with the uncertain/tolerant teacher style, which is in accordance with previous studies [27,43]. Finally, adolescent mental health problems were mildly associated with high levels of depressive symptoms in teachers, although only at primary and senior high school level. The low levels of depressive symptoms in junior high school teachers may have weakened the impact of this factor. Further studies should be carried out to explain the discrepancy.

Several limitations of the present study must be mentioned. First, this study has a cross-sectional design, making it impossible to draw definitive conclusions about the causal relationship between factors. Moreover, adolescent behavior is in a constant stage of development and this study could not determine whether the influences of the classroom persisted over a long-term period or were only transient in duration. Further follow-up and longitudinal studies should be carried out to investigate this question. Second, the results of the present study were based on the responses obtained from adolescents. Class characteristics reported by teachers may be different from those reported by adolescents. Therefore, obtaining data from multiple sources, such as teacher reports and classroom observations, would be useful in future research. Third, despite the significant influence of teacher–student interactions on students’ emotions and behavior, other class factors may have a sizeable impact on the classroom climate and students, such as daily routines and class organization. Similarly, while school-related factors could have been inputted as an additional random component into the multi-level model, this study sought to simplify the analysis by focusing on the classroom and, as such, did not examine such factors. Thus, it was assumed that there was no meaningful variation across schools. Further research should consider these objective indicators and perform a more complex multi-level analysis. Fourth, while such an analysis could include school-level as an additional fixed factor in the model and facilitate direct quantitative comparisons among all levels, this could result in a more intricate and cumbersome model. Thus, the current study conducted separate analyses for primary, junior, and senior high school levels. Finally, a comparison should perhaps not be made between information from non-responders and those responses that produced missing data. Nonetheless, the effective response rate was greater than 75% and the researchers will strive to increase this rate in further studies. Despite these limitations, the researchers believe that these findings, which are based on a fairly large and representative sample, offer some important information regarding Chinese adolescent mental health.

In conclusion, the findings revealed that although the emotional and behavioral problems of Chinese school adolescents showed the strongest association with personality and family environment, the classroom teacher also exerts a significant influence on adolescent mental health. Teacher dissatisfaction was the most influential teacher style for all school-going adolescents. ‘Understanding’ was especially crucial in early adolescence, and ‘helpful/friendly’ was particularly important in late adolescence. These findings can assist in making policy makers more aware of the importance of training teachers to engage in positive communication with students in order to improve adolescent mental health. Special attention should be given to both younger and older adolescents due to their diverse classroom needs.

## Supporting information

S1 Fig The bar of the individual fixed effects on SDQ in each school group. (PDF)

S2 Fig The bar of the class fixed effects on SDQ in each school group. (PDF)

S1 Table Correlations among all the analytical variables used in the regression model in primary school. (PDF)

S2 Table Correlations among all the analytical variables used in the regression model in junior high school. (PDF)

S3 Table Correlations among all the analytical variables used in the regression model in senior high school. (PDF)
