# Supplementary material for: Multilevel analysis of personality, family, and classroom influences on emotional and behavioral problems among Chinese adolescent students
Source: PLoS One. 2018 Aug 9;13(8):e0201442. doi: 10.1371/journal.pone.0201442 (PMC6084894; doi:10.1371/journal.pone.0201442)
Supplement: S1 Fig — (PDF) [file pone.0201442.s001.pdf]

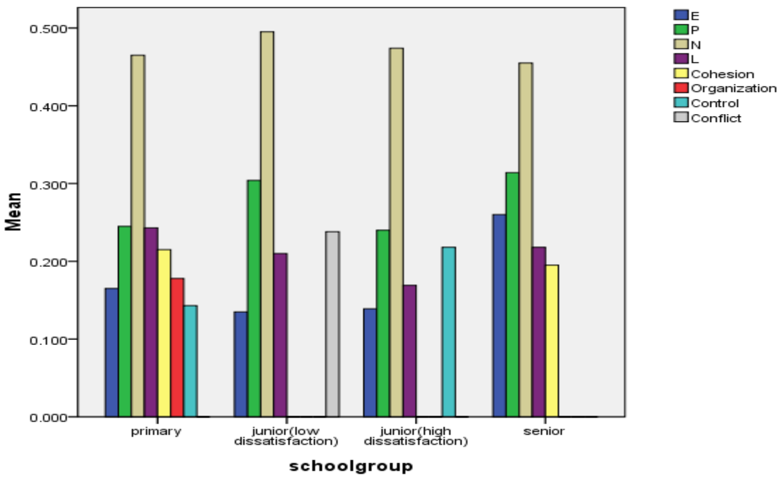

Figure 1. The bar of the individual fixed effects on SDQ in each school group

E: Extraversion; P: Psychoticism; N: Neuroticism; L: Lie
