# Supplementary figures and images for: Multilevel analysis of personality, family, and classroom influences on emotional and behavioral problems among Chinese adolescent students
Source: PLoS One. 2018 Aug 9;13(8):e0201442. doi: 10.1371/journal.pone.0201442 (PMC6084894; doi:10.1371/journal.pone.0201442)

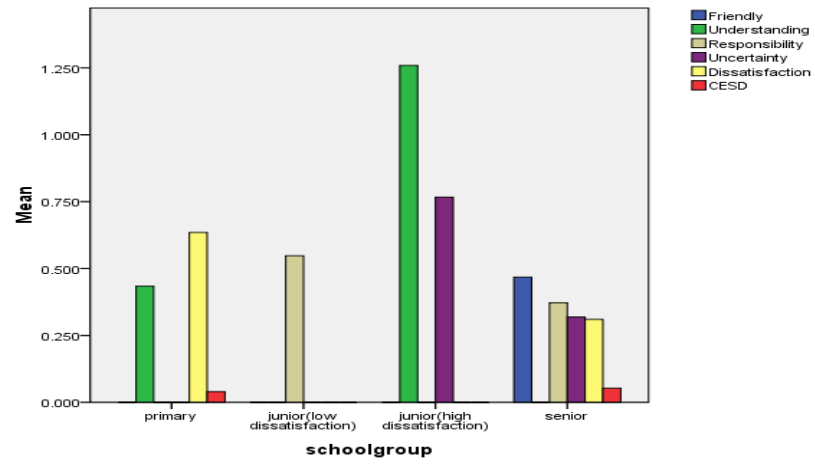

Figure 2. The bar of the class fixed effects on SDQ in each school group  
CESD: Depressive symptoms

Supplement: S2 Fig — (PDF) [file pone.0201442.s002.pdf]
