# Supplementary material for: Multilevel analysis of personality, family, and classroom influences on emotional and behavioral problems among Chinese adolescent students
Source: PLoS One. 2018 Aug 9;13(8):e0201442. doi: 10.1371/journal.pone.0201442 (PMC6084894; doi:10.1371/journal.pone.0201442)
Supplement: S1 Table — (PDF) [file pone.0201442.s003.pdf]

Supplementary Table1 Correlations among all the analytical variables used in the regression model in primary school

	E	P	N	L	F1	F2	F3	F4	F5	F6	F7	Q1	Q2	Q3	Q4	Q5	Q6	Q7	Q8
E	1																		
P	-0.158**	1																	
N	-0.065**	0.581**	1																
L	0.277**	-0.454**	-0.422**	1															
F1	0.323**	-0.430**	-0.329**	0.351**	1														
F2	-0.163**	0.373**	0.364**	-0.325**	-0.529**	1													
F3	0.202**	-0.080**	-0.009	0.086**	0.248**	-0.025	1												
F4	0.260**	-0.146**	-0.205**	0.284**	0.263**	-0.186**	0.119**	1											
F5	0.248**	-0.082**	-0.160**	0.187**	0.186**	-0.127**	0.025	0.547**	1										
F6	0.262**	-0.324**	-0.298**	0.400**	0.554**	-0.364**	0.255**	0.334**	0.207**	1									
F7	0.065**	0.003	-0.062**	0.184**	0.114**	0.021	0.186**	0.204**	0.089**	0.346**	1								
Q1	0.159**	-0.115**	-0.113**	0.145**	0.165**	-0.089**	0.087**	0.155**	0.158**	0.147**	0.019	1							
Q2	0.157**	-0.168**	-0.138**	0.183**	0.208**	-0.144**	0.060**	0.132**	0.121**	0.174**	-0.004	0.616**	1						
Q3	-0.074**	0.217**	0.136**	-0.125**	-0.134**	0.066**	-0.058**	-0.014	0.007	-0.093**	0.006	-0.102**	-0.135**	1					
Q4	-0.032	0.194**	0.211**	-0.196**	-0.133**	0.104**	-0.015	-0.064**	-0.089**	-0.124**	-0.042	-0.117**	-0.316**	0.281**	1				
Q5	0.211**	-0.131**	-0.146**	0.182**	0.226**	-0.161**	0.094**	0.202**	0.194**	0.209**	0.037	0.487**	0.529**	-0.045*	-0.194**	1			
Q6	0.084**	-0.005	-0.054*	0.080**	0.080**	-0.039	0.027	0.125**	0.142**	0.079**	0.000	0.303**	0.301**	0.166**	-0.048*	0.576**	1		
Q7	-0.174**	0.287**	0.211**	-0.271**	-0.243**	0.206**	-0.060**	-0.102**	-0.097**	-0.232**	-0.042	-0.224**	-0.370**	0.348**	0.442**	-0.338**	-0.050*	1	
Q8	0.077**	0.040	0.011	0.001	0.030	-0.008	0.135**	0.048*	-0.005	0.054*	0.081**	0.222**	0.121**	-0.028	0.138**	0.180**	0.054*	0.117**	1
SDQ	-0.304**	0.520**	0.598**	-0.493**	-0.412**	0.346**	-0.095**	-0.240**	-0.158**	-0.374**	-0.056*	-0.188**	-0.213**	0.179**	0.208**	-0.203**	-0.023	0.335**	-0.003

E: Extraversion; P: Psychoticism; N: Neuroticism; L: Lie;
F1 : Cohesion; F2: Conflict; F3: Achievement; F4: Intellectual-Cultural; F5: Active-Recreational; F6: Organization; F7: Control
QI: Leadership; Q2: Understanding ; Q3: Uncertainty; Q4: Admonishing behavior ; Q5: Helpful/Friendly; Q6: Student Responsibility/Freedom ;
Q7: Dissatisfaction ; Q8: Strict behavior;
