# Supplementary material for: Multilevel analysis of personality, family, and classroom influences on emotional and behavioral problems among Chinese adolescent students
Source: PLoS One. 2018 Aug 9;13(8):e0201442. doi: 10.1371/journal.pone.0201442 (PMC6084894; doi:10.1371/journal.pone.0201442)
Supplement: S2 Table — (PDF) [file pone.0201442.s004.pdf]

Supplementary Table2 Correlations among all the analytical variables used in the regression model in junior high school

| | E | P | N | L | F1 | F2 | F3 | F4 | F5 | F6 | F7 | Q1 | Q2 | Q3 | Q4 | Q5 | Q6 | Q7 | Q8 |
|---|---|---|---|---|---|---|---|---|---|---|---|---|---|---|---|---|---|---|---|
| E | 1 | | | | | | | | | | | | | | | | | | |
| P | -0.111** | 1 | | | | | | | | | | | | | | | | | |
| N | -0.088** | 0.578** | 1 | | | | | | | | | | | | | | | | |
| L | 0.201** | -0.452** | -0.417** | 1 | | | | | | | | | | | | | | | |
| F1 | 0.281** | -0.439** | -0.341** | 0.351** | 1 | | | | | | | | | | | | | | |
| F2 | -0.150** | 0.388** | 0.380** | -0.360** | -0.590** | 1 | | | | | | | | | | | | | |
| F3 | 0.173** | -0.077** | -0.050* | 0.158** | 0.287** | -0.113** | 1 | | | | | | | | | | | | |
| F4 | 0.275** | -0.145** | -0.233** | 0.318** | 0.344** | -0.239** | 0.202** | 1 | | | | | | | | | | | |
| F5 | 0.300** | -0.097** | -0.141** | 0.173** | 0.277** | -0.213** | 0.062** | 0.510** | 1 | | | | | | | | | | |
| F6 | 0.218** | -0.321** | -0.352** | 0.430** | 0.531** | -0.425** | 0.284** | 0.362** | 0.223** | 1 | | | | | | | | | |
| F7 | 0.028 | 0.107** | -0.021 | 0.108** | -0.010 | 0.051* | 0.240** | 0.143** | 0.028 | 0.259** | 1 | | | | | | | | |
| Q1 | 0.144** | -0.201** | -0.198** | 0.257** | 0.278** | -0.179** | 0.108** | 0.187** | 0.105** | 0.226** | 0.010 | 1 | | | | | | | |
| Q2 | 0.135** | -0.250** | -0.245** | 0.282** | 0.323** | -0.241** | 0.090** | 0.214** | 0.129** | 0.261** | -0.013 | 0.722** | 1 | | | | | | |
| Q3 | -0.072** | 0.267** | 0.203** | -0.177** | -0.183** | 0.190** | -0.013 | -0.054* | -0.044* | -0.157** | 0.061** | -0.220** | -0.237** | 1 | | | | | |
| Q4 | -0.093** | 0.241** | 0.258** | -0.264** | -0.255** | 0.262** | -0.048* | -0.192** | -0.148** | -0.234** | 0.010 | -0.316** | -0.486** | 0.399** | 1 | | | | |
| Q5 | 0.203** | -0.221** | -0.212** | 0.253** | 0.289** | -0.214** | 0.099** | 0.218** | 0.179** | 0.229** | -0.021 | 0.615** | 0.686** | -0.184** | -0.459** | 1 | | | |
| Q6 | 0.106** | -0.079** | -0.099** | 0.108** | 0.163** | -0.088** | 0.065** | 0.151** | 0.132** | 0.117** | -0.003 | 0.382** | 0.457** | 0.070** | -0.267** | 0.641** | 1 | | |
| Q7 | -0.135** | 0.324** | 0.297** | -0.282** | -0.330** | 0.325** | -0.057** | -0.185** | -0.139** | -0.256** | 0.038 | -0.392** | -0.525** | 0.417** | 0.656** | -0.529** | -0.232** | 1 | |
| Q8 | 0.076** | -0.041 | -0.008 | 0.051* | 0.086** | -0.022 | 0.106** | 0.040 | -0.024 | 0.092** | 0.060** | 0.223** | 0.145** | -0.025 | 0.135** | 0.150** | 0.071** | 0.119** | 1 |
| SDQ | -0.263** | 0.553** | 0.661** | -0.465** | -0.402** | 0.387** | -0.103** | -0.248** | -0.195** | -0.354** | 0.036 | -0.241** | -0.265** | 0.266** | 0.257** | -0.272** | -0.110** | 0.349** | -0.039 |

E: Extraversion; P: Psychoticism; N: Neuroticism; L: Lie;
F1 : Cohesion; F2: Conflict; F3: Achievement; F4: Intellectual-Cultural; F5: Active-Recreational; F6: Organization; F7: Control
QI: Leadership; Q2: Understanding ; Q3: Uncertainty; Q4: Admonishing behavior ; Q5: Helpful/Friendly; Q6: Student Responsibility/Freedom ;
Q7: Dissatisfaction ; Q8: Strict behavior;
