# Supplementary material for: Multilevel analysis of personality, family, and classroom influences on emotional and behavioral problems among Chinese adolescent students
Source: PLoS One. 2018 Aug 9;13(8):e0201442. doi: 10.1371/journal.pone.0201442 (PMC6084894; doi:10.1371/journal.pone.0201442)
Supplement: S3 Table — (PDF) [file pone.0201442.s005.pdf]

Supplementary Table 3 Correlations among all the analytical variables used in the regression model in senior high school

	E	P	N	L	F1	F2	F3	F4	F5	F6	F7	Q1	Q2	Q3	Q4	Q5	Q6	Q7	Q8
E	1																		
P	-0.096**	1																	
N	-0.042	0.470**	1																
L	0.096**	-0.375**	-0.384**	1															
F1	0.207**	-0.384**	-0.322**	0.263**	1														
F2	-0.098**	0.313**	0.410**	-0.274**	-0.580**	1													
F3	0.152**	-0.052	-0.022	0.185**	0.243**	-0.093**	1												
F4	0.206**	-0.032	-0.213**	0.282**	0.271**	-0.218**	0.207**	1											
F5	0.297**	-0.052	-0.178**	0.137**	0.253**	-0.143**	0.128**	0.514**	1										
F6	0.212**	-0.230**	-0.335**	0.384**	0.451**	-0.364**	0.311**	0.350**	0.257**	1									
F7	0.021	0.115**	-0.041	0.168**	0.019	-0.036	0.220**	0.232**	0.082**	0.337**	1								
Q1	0.109**	-0.290**	-0.214**	0.214**	0.300**	-0.183**	0.151**	0.111**	0.101**	0.251**	0.013	1							
Q2	0.084**	-0.317**	-0.240**	0.214**	0.305**	-0.192**	0.125**	0.106**	0.080**	0.215**	-0.015	0.736**	1						
Q3	-0.068*	0.251**	0.146**	-0.123**	-0.187**	0.097**	-0.062*	-0.058*	-0.031	-0.144**	0.048	-0.341**	-0.342**	1					
Q4	-0.014	0.226**	0.235**	-0.234**	-0.212**	0.200**	-0.045	-0.135**	-0.059*	-0.176**	-0.050	-0.360**	-0.558**	0.410**	1				
Q5	0.080**	-0.237**	-0.223**	0.213**	0.295**	-0.204**	0.153**	0.208**	0.133**	0.226**	0.028	0.621**	0.681**	-0.216**	-0.516**	1			
Q6	-0.014	-0.098**	-0.136**	0.138**	0.166**	-0.126**	0.095**	0.167**	0.087**	0.127**	0.072**	0.419**	0.486**	0.040	-0.341**	0.674**	1		
Q7	-0.108**	0.362**	0.197**	-0.197**	-0.326**	0.232**	-0.070*	-0.090**	-0.077**	-0.192**	0.062*	-0.402**	-0.544**	0.434**	0.655**	-0.526**	-0.257**	1	
Q8	0.094**	-0.079**	-0.070*	0.121**	0.083**	-0.055*	0.094**	0.043	0.049	0.157**	0.074**	0.298**	0.167**	-0.055*	0.139**	0.151**	0.035	0.079**	1
SDQ	-0.275**	0.518**	0.626**	-0.415**	-0.389**	0.360**	-0.089**	-0.183**	-0.176**	-0.352**	-0.031	-0.253**	-0.269**	0.243**	0.244**	-0.226**	-0.084**	0.307**	-0.095**

E: Extraversion; P: Psychoticism; N: Neuroticism; L: Lie;
F1 : Cohesion; F2: Conflict; F3: Achievement; F4: Intellectual-Cultural; F5: Active-Recreational; F6: Organization; F7: Control
QI: Leadership; Q2: Understanding ; Q3: Uncertainty; Q4: Admonishing behavior ; Q5: Helpful/Friendly; Q6: Student Responsibility/Freedom ;
Q7: Dissatisfaction ; Q8: Strict behavior
